# Respiratory Health in Waste Collection and Disposal Workers

**DOI:** 10.3390/ijerph13070631

**Published:** 2016-06-24

**Authors:** Luigi Vimercati, Antonio Baldassarre, Maria Franca Gatti, Luigi De Maria, Antonio Caputi, Angelica A. Dirodi, Francesco Cuccaro, Raffaello Maria Bellino

**Affiliations:** 1 Interdisciplinary Department of Medicine, Occupational Medicine “B. Ramazzini”, University of Bari Medical School, Bari 70124, Italy; mariafranca.gatti@gmail.com (M.F.G.); luidemale@gmail.com (L.D.M.); anto.caputi@gmail.com (A.C.); angelica.dirodi@gmail.com (A.A.D.); 2 Health Local Unit of Barletta-Andria-Trani, Barletta 76121, Italy; francesco_cuccaro@hotmail.com; 3 Health Local Unit of Bari, Bari 70122, Italy; raffaello.bellino@gmail.com

**Keywords:** occupational exposure, bioaerosol, endotoxins, occupational respiratory disease, waste workers

## Abstract

Waste management, namely, collection, transport, sorting and processing, and disposal, is an issue of social concern owing to its environmental impact and effects on public health. In fact, waste management activities are carried out according to procedures that can have various negative effects on the environment and, potentially, on human health. The aim of our study was to assess the potential effects on respiratory health of this exposure in workers in the waste management and disposal field, as compared with a group of workers with no occupational exposure to outdoor pollutants. The sample consisted of a total of 124 subjects, 63 waste collectors, and 61 office clerks. Informed consent was obtained from all subjects before inclusion in the study. The entire study population underwent pulmonary function assessments with spirometry and completed two validated questionnaires for the diagnosis of rhinitis and chronic bronchitis. Statistical analyses were performed using STATA 13. Spirometry showed a statistically significant reduction in the mean Tiffenau Index values in the exposed workers, as compared with the controls, after adjusting for the confounding factors of age, BMI, and smoking habit. Similarly, the mean FEV1 values were lower in the exposed workers than in the controls, this difference being again statistically significant. The FVC differences measured in the two groups were not found to be statistically significant. We ran a cross-sectional study to investigate the respiratory health of a group of workers in the solid waste collection and disposal field as compared with a group of office workers. In agreement with most of the data in the literature, our findings support the existence of a prevalence of respiratory deficits in waste disposal workers. Our data suggest the importance of adopting preventive measures, such as wearing specific individual protection devices, to protect this particular category of workers from adverse effects on respiratory health.

## 1. Introduction

Waste management, namely, collection, transport, sorting and processing, and disposal, is an issue of social concern owing to its environmental impact and effects on public health. In fact, waste management activities are carried out according to procedures that can have various negative effects on the environment and, potentially, on human health. They pose risks resulting from the emissions or release of hazardous chemical agents and biological agents, from the types of exposure to these agents, and from the susceptibility of the populations exposed to them.

Epidemiological studies of the short- and long-term effects of exposure to waste on public health have been focused above all on identifying any associations between residence in the vicinity of a landfill and adverse effects on health [1,2,3]. Meanwhile, less attention has been paid to the risk posed in subjects who are directly involved in waste management, especially as regards the effects on the respiratory tract. Workers in the waste disposal field, in particular, can be exposed to bioaerosols, gases, and vapours.

Waste sorting, collection, and recycling, procedures that have now been adopted by nearly all EU member states, have actually introduced new risk profiles for garbage collectors, largely attributable to the inhalation of bioaerosols generated by the decomposition of organic waste [4,5]. This process can lead to the formation of various biological agents including bacteria, fungi, and volatile compounds such as endotoxins, β(1-3)-glucans, and mycotoxins that can provoke inflammation of the airways [6,7,8].

In particular, endotoxins, the components of the cell wall of Gram-negative bacteria, are considered to be the most powerful proinflammatory component present in bioaerosols [9]. Exposure to low concentrations of endotoxins seems to induce an inflammatory response of the upper airways, through neutrophil activation and the release of cytokines such as IL6 and IL8 and TNF-alpha, the main factors triggering inflammatory processes [10,11]. The levels of these proinflammatory mediators were found to be increased in patients with a diagnosis of chronic obstructive respiratory diseases [12]. β(1-3)-glucans are an integral part of the wall of fungal spores, but are also present in some bacteria. Experimental studies in animals have shown that the inflammatory response is characterised by an increased number of eosinophils [13] and that repeated exposure to β(1-3)-glucans exacerbates the inflammatory response to endotoxins via a synergic action [14].

Exposure to bioaerosols, therefore, seems to induce an inflammatory response at the level of the airways mucosa, probably due to a complex interaction among microorganisms or cell wall components of these microorganisms and the host immune system [5].

When assessing the respiratory health risk in solid waste disposal workers, it is also important to take into account the physical effort and muscle work that these activities entail, during lifting and manhandling of the various containers. This induces a ventilation response consisting of an increased flow volume and respiratory frequency [15,16] that will increase the amount of bioaerosol inhaled, including not only airborne organic compounds, but also dust particles and vehicle exhaust fumes and gases. These factors have been reported to be responsible for a higher incidence of respiratory diseases in this worker category [6,7,17,18,19], namely, influenza-like disorders (rhinitis, conjunctivitis, cough, headache) [20], as well as more severe obstructive disorders such as chronic obstructive bronchopneumonia [21] and allergic bronchopulmonary aspergillosis [22,23], and in some cases restrictive lung diseases, which are especially related to the exposure to elements or chemicals such as Al, Si, carbon black, TiO_2_, silicon oxide, talcum powder, asbestos, and other fibres [24].

The aim of our study was to assess the potential effects on respiratory health of this exposure in workers in the waste management and disposal field, as compared with a group of workers with no occupational exposure to outdoor pollutants.

## 2. Materials and Methods

The research was carried out on a working population of 375 employees of two waste collection and disposal companies, 300 waste collectors (exposed), and 75 clerks (controls). Both exposed and control subjects were randomly selected to reduce the possibility of bias in self-selection. Upon inclusion in the study, a completed questionnaire as regards medical and working history, as well as various confounding factors, was collected from all workers in the presence of a physician.

**Figure 1:**
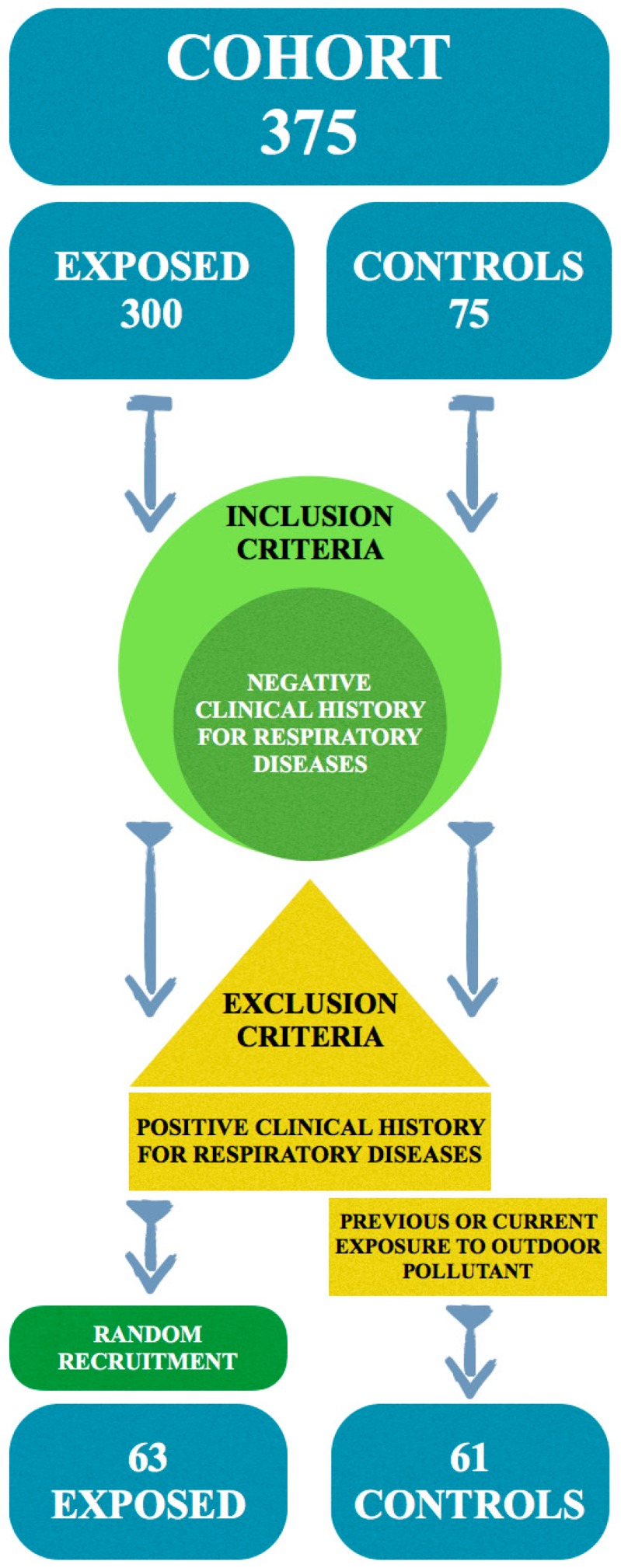
Participation and recruitment flow chart.

The response rate was higher for exposed (94%) than for controls (91%). All recruited subjects, both exposed and non-exposed, had a negative clinical history for previous respiratory diseases (inclusion criteria); only for non-exposed subjects did we adopt as exclusion criteria previous or current occupational exposure to outdoor pollutants. The final sample consisted of a total of 124 subjects, 63 exposed and 61 controls (Figure 1). The investigation was carried out over a period from July to November 2013, and the two groups were studied at the same time to eliminate any confounding factor introduced by seasonal variations. The waste disposal activities of the exposed workers were carried out 5 days a week for 6 h/day and consisted of emptying different-sized containers (wheely-bins, dumpsters) manually or mechanically with waste compactors, sometimes with ground assistance from the mechanised refuse collection service. Informed consent was obtained from all subjects before inclusion in the study. All subjects agreed to the processing of their personal data, treated as sensitive data. All subjects were informed that data from the research protocol would be processed anonymously and collectively, applying proper scientific methods and for scientific purposes, in accordance with the principles of the Helsinki Declaration. Our study is in accordance with good clinical practice guidelines. Both spirometric and questionnaire were performed in agreement with the health surveillance protocol and health promotion’s campaigns (Italian Legislative Decree N° 81 of 2008, in transposition of several European Directives).

All the recruited subjects then completed two questionnaires validated for the diagnosis of rhinitis [25] and chronic bronchitis [26]. The first one probed for the presence, frequency, and severity of rhinitis symptoms like rhinorrhoea, sneezing, nasal congestion, and pruritus, while the second was aimed at diagnosing chronic bronchitis, verifying whether the subject suffers from coughing, catarrh, dyspnoea, or other symptoms suggesting bronchial asthma, such as wheezing, coughing up mucous, and a feeling of chest oppression. Rhinitis symptoms definition was based on the International Chronic Rhinitis (ICR) working group recommendation: the occurrence of two or more symptoms (nasal obstruction, rhinorrhea, sneezing, or itchy nose) on most days during the past year. Chronic bronchitis symptoms definition is based on the occurrence of cough that lasts for at least 3 months, 2 years in a row, associated with catarrh, dyspnea, wheezing, and a feeling of chest oppression. The entire study population then underwent pulmonary function assessments. Spirometry was performed with the Pony FX spirometer (version 1.7, COSMED srl, Albano Laziale, Rome, Italy) to assess the respiratory function and to identify any obstructive or restrictive deficits.

All respiratory function tests were performed by the same operator, with the subject in a sitting position with his nose closed by a clip, following the American Thoracic Society (ATS) guidelines. The pulmonary function test was performed three times for each subject, and we chose the best one. The most important aspects of spirometry are the forced vital capacity (FVC), which is the volume delivered during an expiration made as forcefully and completely as possible starting from full inspiration, and the forced expiratory volume (FEV1) in one second, which is the volume delivered in the first second of an FVC manoeuvre. Spirometric airflow limitations were defined according to the Tiffenau Index as (FEV1/FVC) < 70% or an FEV1 < 80% of predicted values. A restrictive ventilatory pattern is characterised by a proportional reduction in FEV1 and FVC < 80% with a normal FEV1/FVC [27]. Statistical analyses were performed via analysis of variance (ANOVA) to seek statistically significant differences between the exposed group (waste disposal workers) and the non-exposed group (office workers) for the continuous variables, after adjusting the models for confounding factors (age, BMI, and smoking habit). Other confounding factors, such as socioeconomic status (SES) and educational level were not considered relevant, because all participants belonged to classes IIIM (skilled manual—exposed) or IIINM (skilled non-manual—controls) and had completed secondary education. Only three participants, among the controls, had a university degree. We did not study the confounding interaction of cardiovascular diseases. Logistic regression models were applied to assess dichotomous spirometric and symptoms variables (rhinitis and bronchitis) related to exposure, again after adjusting for the same confounding factors. Statistical analyses were performed using STATA version 13 Software (STATA Corporation, College Station, TX, USA).

## 3. Results

All 124 study participants were male Caucasians (mean age 53 years for exposed workers and 51 for controls) and worked in the Apulia region in Southern Italy. As regards smoking habits, a confounding factor, 50.8% of exposed subjects were smokers or formerly smokers, while only 37.7% of controls had a history of smoking. Characteristics of both groups are shown in Table 1. The pulmonary function test was performed three times for each subject, and we chose the best one. Spirometry showed, as reported in Table 2, a statistically significant reduction in the mean Tiffenau Index values in the exposed workers (75.08%) compared with the controls (79.93%) after adjusting for the confounding factors of age, BMI, and smoking habit. Similarly, the mean FEV1 values were lower in the exposed workers (3.53 L) than in the controls (3.64 L), this difference again being statistically significant. The FVC differences measured in the two groups were not found to be statistically significant (controls, 4.56; exposed, 4.76). The exposed workers also showed more spirometric alterations, which were statistically significant, compared with the non-exposed group (OR = 8.42; 95% CI: 1.8–38.9), again after adjusting for age, BMI, and smoking habit (OR = 7.9; 95% CI: 1.7–37.0), as reported in Table 3. When analysing the questionnaires on respiratory symptoms, rhinitis symptoms were found to be prevalent in the non-exposed group (31.1%) as compared with the exposed subjects (17.4%) (OR = 0.41; 95% CI: 0.17–1.00), whereas lower airway obstruction symptoms, investigated in the questionnaire for diagnosing bronchitis, were slightly more prevalent in the exposed group (19.0%) than in the controls (18.0%), as shown in Table 4. In both cases, the results were not statistically significant, even after adjusting for the confounding factors.

## 4. Discussion

We ran a cross-sectional study to investigate the respiratory health of a group of workers in the solid waste collection and disposal field as compared with a group of office workers. The spirometric tests performed showed a significant reduction in the Tiffenau Index and FEV 1 values in the exposed workers, after adjusting for the confounding factors. Meanwhile, no statistically significant differences were found among the FVC values. These results suggest the prevalence of an obstructive pulmonary pattern and seem to be in agreement with the pathogenic hypothesis whereby cytokines, such as IL6, IL8, and TNF-alpha, which are produced as a result of contact with bacterial endotoxins of bioaerosols, may be the main mediators of the inflammatory response involved in the development of chronic obstructive lung diseases. This finding is in agreement with the results obtained by Athanasiou et al., who demonstrated a statistically significant reduction in FVC and FEV1 in a group of 104 municipal solid waste disposal workers in the city of Keratsini (Grecia) compared with a control group of 80 office workers [28]. Comparable results were obtained in a study conducted in India in 96 solid waste disposal workers at an open landfill. A significant reduction in FEV1 and the Tiffenau Index values was found in the exposed workers compared with the controls [15].

In our study, the results of the allergological questionnaires did not reveal statistically significant differences between the two study populations, even if we found a higher prevalence of rhinitis symptoms in the controls, related to possible indoor air pollution. This evidence is not in agreement with Athanasiou et al. who showed that respiratory symptoms, investigated by means of a validated questionnaire, were also significantly more common in the exposed group than the control group. Moreover, in the exposed group studied by Ray et al. [15], there was a statistically significant prevalence of respiratory symptoms. We did not find differences in the allergological questionnaires because our control group is represented by office clerks. For these subjects, we can assume a possible exposure to indoor pollutants, such as those emitted from office equipment (volatile organic chemicals (VOCs), ozone, and particulate matter), biological contaminants (bacteria, virus, fungi including moulds), and other chemicals (office furniture, cleaning, and consumer products).

In a recent study made in 25 waste handlers at a landfill near Oslo, the spirometric FEV1 values were significantly lower at the end of the workweek than at the beginning of the week. In addition, cytological examination of sputum showed an increased percentage of neutrophils and IL-8, supporting the hypothesis that an etiopathogenic mechanism of an aspecific inflammatory nature, mediated by neutrophils, underlies the spirometric alterations [5].

In a study by Gea de Meer, only a slight reduction in FEV1 was observed at the end of the workweek in a group of 16 organic waste loaders, only 6 of whom had a positive history for respiratory symptoms, which was determined by questionnaire. The bronchial challenge test subsequently performed with methacholine showed a significantly reduced FEV1 in the symptomatic subjects only. The authors concluded that the occupational exposure could be responsible for exacerbating airway inflammation only in those subjects with preexisting respiratory symptoms [29].

In agreement with most of the data in the literature, our findings support the existence of a prevalence of respiratory deficits, as evidenced by spirometry, in waste disposal workers. Although this conclusion is not based on a correlation with the respiratory symptoms investigated in the questionnaires, it seems plausible to assume that the altered ventilatory function parameters obtained in our study may be considered as a preclinical indicator of respiratory diseases in subjects who have not yet shown the onset of clinical symptoms.

It should be noted that our study, which was not conducted in a very large population, did not include environmental monitoring data on the concentrations of allergens, endotoxins, dust, and toxic substances in the areas surrounding the exposed group’s work zones. In particular, it would be useful to measure the concentration of endotoxins and β(1-3)-glucans that seem to play a key role, even via a synergic action, in inducing an inflammatory response of the upper airways. However, it is reasonable to suppose that there will be no significant differences in the concentrations values measured in other European nations with comparable waste management policies compared to those adopted in Italy [30]. It must also be borne in mind that waste collection, like other prevalently outdoor working activities, can expose workers to urban pollutants (i.e., particulate matter, nitrous oxide, carbon dioxide, ozone, and carbon monoxide) [31,32,33], known to play a causal or concausal role in the onset of respiratory symptoms and spirometric alterations.

Our data, which need to be confirmed with larger samples, suggest the importance of adopting preventive measures such as wearing specific individual protection devices, to protect this particular category of workers from adverse effects on respiratory health.

## 5. Conclusions

Waste management is an issue of social concern owing to its environmental impact and effects on public health. In fact, waste management activities are carried out according to procedures that can have various negative effects on the environment and, potentially, on human health. On this basis, we ran a cross-sectional study to evaluate respiratory health effects in a group of workers in the solid waste collection and disposal field, compared with a group of administrative employees. Our study showed a significant reduction in the Tiffenau Index and FEV 1 values in the exposed workers as compared to the controls, after adjusting for confounding factors. These data suggest the importance of adopting preventive measures, such as wearing specific Personal Protection Equipments (PPEs), to protect this category of workers from adverse effects on respiratory health.

## Figures and Tables

**Table 1:** Characteristics of participants.

Characteristics		Exposed	Controls	*p* Values
*N*		63	61	
Sex	M	63	61	
	F	0	0	
Age	Average	53	51	0.38
	SD	7	7	
	Median	54	52	
	Range	32–66	36–63	
BMI	Average	26.40	26.00	0.71
	SD	11.06	4.36	
	Median	26.37	25.25	
	Range	16.90–34.84	18.36–40.14	
Smoking habit	Yes	17 (27.00%)	13 (21.30%)	0.19
	No	31 (49.20%)	38 (62.30%)	
	Former	15 (23.80%)	10 (16.40%)	

SD = Standard deviation; Significance *p* < 0.05.

**Table 2:** Spirometric test results.

Spirometric Values		Exposed	Controls	*p* Values
*N*		63	61	
FVC	Average	4.76	4.56	0.114
	SD	0.99	1.09	
	Median	4.67	4.49	
	% mean	115.06	114.2	
	% SD	22.42	18.06	
	% median	113	112	
FEV1	Average	3.53	3.64	0.046
	SD	0.73	0.85	
	Median	3.42	3.50	
	% mean	105.89	111.03	
	% SD	19.05	17.53	
	% median	107	110	
TIFFENAU INDEX	Average	75.08	79.93	0.001
	SD	9.06	3.89	
	Median	76.09	80.12	
	Range	32.62–93.43	67.57–87.31	

SD = Standard deviation; Significance *p* < 0.05.

**Table 3:** Association between occupational exposure to waste and spirometric alterations using logistic regression.

OR	95% CI	*p* Value
8.4	1.8–38.9	0.006
7.9 *	1.7–37.0	0.008

* Adjusted for age, BMI, and smoking habit.

**Table 4:** Association between occupational exposure to waste and symptoms in questionnaire.

Symptoms	Exposed (*N* = 63)		Controls (*N* = 61)		OR	95% CI	*p* Value
	*N*	%	*N*	%			
Rhinitis symptoms	11	17.4	19	31.1	0.41	0.17–1.00	0.051
Bronchitis symptoms	12	19.0	11	18.0	1.09	0.43–2.76	0.847
